# Talent Development in Young Cross-Country Skiers: Longitudinal Analysis of Anthropometric and Physiological Characteristics

**DOI:** 10.3389/fspor.2020.00111

**Published:** 2020-10-19

**Authors:** Chiara Zoppirolli, Roberto Modena, Alessandro Fornasiero, Lorenzo Bortolan, Spyros Skafidas, Aldo Savoldelli, Federico Schena, Barbara Pellegrini

**Affiliations:** ^1^CeRiSM (Sport Mountain and Health Research Center), University of Verona, Verona, Italy; ^2^Department of Neuroscience, Biomedicine and Movement Sciences, University of Verona, Verona, Italy

**Keywords:** adolescence, performance, physiological indicators, talent selection, age

## Abstract

**Introduction:** Very little is known about talent development and selection processes in young cross-country skiers.

**Aim:** (1) to analyze the effect of age on anthropometric and physiological parameters in medium-to-high level cross-country skiers during the late teenage period; (2) to describe parameters' trend in selected talents after the late teenage period; (3) to define which characteristics during the late teenage period could discriminate against further talent selection.

**Method:** We found 14 male (M) and nine (F) athletes in our database, identified as talents by regional teams during the late teenage period, who performed the same diagonal-stride roller-skiing incremental test to exhaustion at 17 and 18 years old. Of these, four M and three F teenagers performed four further evaluations, and were selected by the national team. Age effect during the late teenage period was verified on anthropometric and physiological parameters measured at maximal intensity (MAX), first (VT1), and second (VT2) ventilatory thresholds, and 3° and 6° of treadmill incline. An observational analysis allowed to evaluate parameters' trend after the late teenage period in selected athletes, and to determine possible characteristics early discriminating further selection.

**Results:** During the late teenage period, height, weight, and BMI was still raising in M as well as V'O_2_ at VT2 and 6° of treadmill incline (all *P* > 0.05). In F, mass-scaled V'O_2_ MAX increased while heart rate (HR) at MAX and VT2 decreased (all *P* > 0.05). Since the late teenage period, all selected males showed maximal ventilation volumes, absolute V'O_2_ at MAX, VT1, and VT2 that were within or above the 75th percentile of their group; the same was found in selected females for mass-scaled V'O_2_ MAX, VT1, and VT2 time. After the late teenage period, all selected athletes showed an increasing trend for VT2 time, while a decreasing trend for sub-maximal energetic cost, %V'O_2_ and HR.

**Discussion:** During the late teenage period, males are still completing their maturation process. Since the late teenage period, some physiological parameters seem good indicators to early discriminate for further talents. A progressive increase in skiing efficiency was demonstrated in developing talents of both sexes after the late teenage period.

## Introduction

Cross-country (XC) skiing has received much attention in last few decades and the number of investigations addressing the many aspects related to this sport discipline has grown incredibly. Scientific literature provides a rich and updated panorama about the physiological characteristics of adult skiers from high to top performance levels, as well as the determinants of performance in this sport (Eisenman et al., 1989; Hoffman and Clifford, 1992; Holmberg, 2015; Hebert-Losier et al., 2017; Sandbakk and Holmberg, 2017; Losnegard, 2019; Zoppirolli et al., 2020). It emerged that best adult XC skiers present exceptionally high sport-specific maximal oxygen consumption (V'O_2_ MAX) values (between 80 and 90 and between 70 and 80 mL·min·kg^−1^· min^−1^ for men and women, respectively) ventilation volumes (higher than 230 L·min^−1^), anaerobic capacity, upper-body strength, and power (Eisenman et al., 1989; Hoffman and Clifford, 1992; Hebert-Losier et al., 2017; Sandbakk and Holmberg, 2017; Losnegard, 2019) as well as skiing efficiency (Sandbakk et al., 2010a,b).

On the other hand, less is known about the physiological characteristics of young XC skiers aged between 17 and 20 years. It was demonstrated that the physiological intensity sustained during XC skiing races lasting < 25 min equals the onset of blood lactate accumulation (OBLA) intensity (considered as 4 mMol·L^−1^ of blood lactate concentration) in junior female skiers aged between 17 and 19 years (Welde et al., 2003). It is also known that around 18 years old, sprint (Sandbakk et al., 2011) to mid-distance (Larsson et al., 2002) XC skiing performance is related to absolute running V'O_2_ MAX, as well as to the absolute oxygen consumption at ventilatory thresholds and OBLA intensities (Larsson et al., 2002) in male athletes, while it is related to physiological parameters at unitary respiratory quotient in females (Larsson et al., 2002). Raising the subjects' OBLA by emphasizing the volume of polarized training has been suggested to be of particular importance for these athletes (Welde et al., 2003; Sandbakk et al., 2011). Additionally, roller-skiing time trials using double-poling, diagonal-stride, or running (around 6, 10, and 10 min, respectively) are accurate predictors of both sprint and distance skiing performance (the two rankings being strongly correlated), for both male and female junior XC skiers of 18 years of age (Carlsson et al., 2014).

Even less is known on younger athletes, although they are encouraged to train intensely from an early age. For XC skiing it was suggested that during puberty, maturity offset (*i.e.*, time distance from peak height velocity at the moment of the athlete' performance evaluation) is an important confounding factor that influences the majority of the existing relationships between XC skiing performance and physical skills (Stöggl et al., 2015, 2017). For instance, it was shown that the correlations between XC skiing performance and the various physical skills in male athletes are reduced or even annulled when maturity offset is entered as a covariate in the data analysis (Stöggl et al., 2015, 2017).

Very little has been published about the longitudinal evolution of physiological parameters in young XC skiers, as well as about the talent recognition process, from detection to selection (Williams and Reilly, 2000). To the best of our knowledge, only two early longitudinal studies reported the development of V'O_2_ MAX and anaerobic threshold during adolescence in well-trained athletes (Rusko, 1987, 1992). It was shown that V'O_2_ MAX increases from pre-puberty to adolescence with a trend to level-off after 20 years of age, and with international level XC skiers being able to further improve even after the teenage period. Moreover, the percentage of V'O_2_ MAX measured at anaerobic threshold tended to be constant over time in those athletes (Rusko, 1987, 1992). On the other hand, in the current literature, anthropometric or physiological parameters of young XC skiers that can possibly predict further success in XC competitions have not yet been identified.

Concerned the process of talent identification/selection in sport activities, it was suggested that an evaluation of the “current status” of athletes might increase the risk of false positives or negatives (Pickering and Kiely, 2017), because of the influence of maturation state, individual trainability, and residual trainability after the teenage period. This approach was suggested to promote the selection of high-performing individuals at the present time, rather than identifying those individuals with the greatest capacity to improve. An “*a posteriori*” approach, based on the comparison of data from different groups of young athletes that subsequently had further success, seemed to be a valid alternative strategy in the process of talent identification or selection (Pickering and Kiely, 2017), that might also include XC skiing.

Thus, the aims of our investigation were (i) to analyze the effect of age on anthropometric and physiological characteristics measured during the late teenage period (well over the occurrence of peak maturation velocity) in XC skiers already identified by regional teams, (ii) to describe the trend of the measured parameters in selected talents after the late teenage period, in the athletes selected by the national team; (iii) to observe “*a posteriori”* which characteristics during the late teenage period might help with further talent selection, by discriminating who was subsequently selected by the national team from who was not. According to previous investigations, we hypothesized the effect of age on the anthropometric characteristics of male athletes during the late teenage period as well as a potential role of maximal oxygen consumption, ventilation, and occurrence of ventilatory thresholds in discriminating talented XC skiers since their late teenage period. Moreover, we hypothesized that also skiing economy, as a measure of the technical skills, would discriminate future performance levels in adolescent XC skiers.

## Methods

The study design was planned as a longitudinal and observational research.

### Inclusion Criteria for Data Analysis

**Figure 1:**
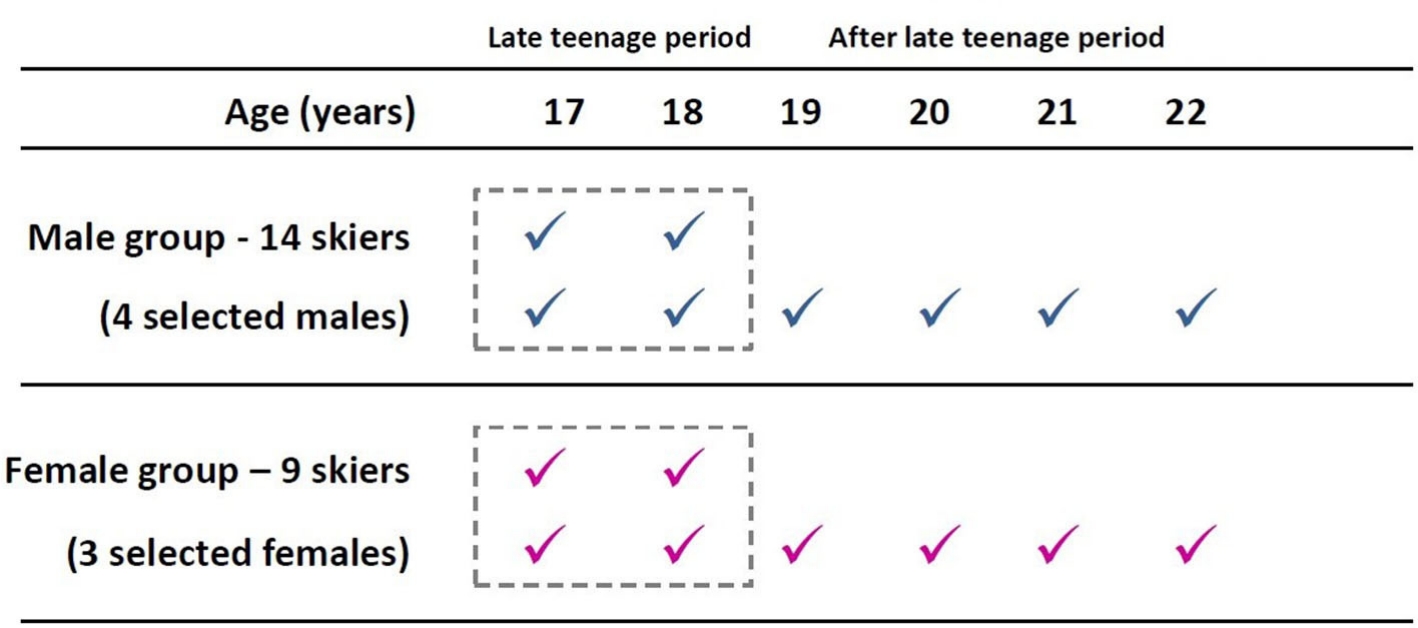
Visual overview of the longitudinal research. The figure shows the time-course of the tests yearly performed by male (blue) and female (purple) athletes. All the athletes performed two consecutive evaluations from 17 to 18 years of age, while only the selected athletes did six consecutive evaluations from 17 to 22 years of age. Late teenage (dotted-lined boxes) and after late teenage periods were here represented.

We searched our database for Italian male (M) and female (F) XC skiers that (i) had come into our laboratories for testing purposes since the age of 17, as members of XC skiing regional teams or Italian national team, (ii) had performed the same diagonal-stride roller-skiing protocol for the evaluation of maximal physiological parameters and ventilatory thresholds (iii) had skied on the same treadmill and with the same roller-skiing technique, (iv) had skied for consecutive years [at least 2 for skiers identified by the regional teams only, at least 6 for the skiers further selected by the National team (Figure 1)], (v) had been tested between October and November (to monitor the same period of training periodization) from 2008 to 2019.

### Testing Protocol, Instruments, and Materials

All the skiers observed at least 48 h of low-intensity training before testing, free hydration was allowed. Before testing, athletes' weight and height were measured in underwear by a digital scale with a resolution of 0.1 kg, and barefooted through a stadiometer with 0.001 m resolution, respectively. Roller-skiing testing procedures were performed in the same laboratory, with temperature and humidity being kept constant during the test sessions (i.e., 18°C and 60% rH, respectively).

The roller skies testing protocol (designed in agreement with coaches since 2007) consisted of (i) 10 min warm-up with the diagonal-stride technique at 2° of treadmill slope and 10 or 9 km·h^−1^, for M and F, respectively—this procedure induced a similar warm-up of roller-skis' wheels, thus reducing rolling friction coefficient differences among skiers; (ii) 10 min rest during which athletes were equipped with heart rate monitor and mask for metabolic measurements; (iii) an incremental test performed consistently with the diagonal-stride technique, starting from the mechanical intensity of the warm-up and increasing the treadmill slope by 1° every 3 min, until voluntary exhaustion.

All the tests analyzed in the present investigation were performed on the same 2.5 × 3.5-m motor-driven treadmill (Rodby Innovation AB, Vänge, Sweden). The athletes always used their own ski boots, while poles and roller-skies were provided by the lab. The poles (ONE WAY Sport Oy, Helsinki, Finland) were equipped with special tips designed not to slip on the treadmill belt surface during arm poling and were available at multiple lengths with 2.5 cm differences. The athletes were asked to use their usual pole length taken during classic competitions. The roller-skies used across several years of testing (Ski Skett Nord CL, Crestani Sport, Sandrigo, Italy) were the same as far as concerns the metallic structure, while the wheels were regularly changed for new wheels of identical characteristics every 100 h of usage, due to rubber consumption. However, friction coefficient measured after each wheel change (Pellegrini et al., 2011) ranged from 0.0230 to 0.0237, ensuring a constant friction coefficient across the different testing sessions.

During the maximal incremental test to exhaustion, ventilatory parameters were continuously collected by a breath-by-breath metabolic cart. Across the several years of testing, two metabolic carts of the same company have followed each other, due to the renewal of the laboratory equipment (Quark b2, Cosmed, Rome, Italy and C-PET, Cosmed, Rome, Italy). In both cases, the athletes wore an appropriately sized facial mask (70 mL dead space) to direct the respiratory flows coming from mouth and nose to the air gas analyzers, through a sampling tube. Ventilatory volumes were measured by the optic reading of the movement velocity of a low-resistance bidirectional turbine incorporated in the mask. Before each test, the gas analyzers and the turbine were calibrated according to manufacturing guidelines with ambient air (20.93% for oxygen and 0.03% for carbon dioxide), a gas tank with a known concentration of gasses (16.00 ± 0.04% oxygen and 5.00 ± 0.01% carbon dioxide) (Air Liquide Italia S.p.A., Milan, Italy), and a 3-L volume syringe.

At the end of each 3-min stage, the skiers stopped poling with the left upper-limb so that a sample of peripheral blood could be taken from the fingertip and collected in a 25-μL capillary tube. A sample of blood was also taken at the end of the test. Blood lactate concentration was measured using a digital blood lactate analyzer (Biosen C-line, EKF Diagnostic, GmbH, Magdeburg, Germany). Heart rate was measured continuously during the test with a wireless monitoring system (Polar Electro Oy, Kempele, Finland) connected to the metabolic cart.

First (VT1) and second (VT2) ventilatory thresholds were detected by visual inspection of two independent researchers familiar with testing reporting. VT1, considered as an indicator of lactate accumulation, was determined by analyzing a group of different measures including: (i) the first disproportionate increase in ventilation; (ii) an increase in VE/VO_2_ with no increase in VE/VCO_2_; and iii) an increase in end-tidal O_2_ tension with no consequent fall in end-tidal CO_2_ tension (Reinhard et al., 1979; Wasserman et al., 1990). VT2, considered as an indicator of metabolic acidosis development, was determined by detecting (i) the second disproportionate increase in minute ventilation; (ii) the first systematic increase in VE/VCO_2_; and (iii) the first systematic decrease in end-tidal CO_2_ tension (Reinhard et al., 1979; Wasserman et al., 1990). Maximal physiological parameters were defined as the highest value after 20-s of data smoothing (Robergs et al., 2010), while maximal lactate concentration was measured at the end of the test. The physiological values relative to each test stage were considered as the average values of the last 30 s of each stage, after 5-s of data smoothing (Sandbakk et al., 2010a; Zoppirolli et al., 2015).

### Parameters Analyzed

Weight, height, and BMI were analyzed as anthropometric parameters. Maximal absolute and mass-scaled oxygen consumption (V'O_2_ MAX and V'O_2_ MAX ·kg^−1^, respectively), ventilation (V' MAX), heart rate (HR MAX), maximal blood lactate concentration (Blood Lactate MAX), and time to exhaustion (Time MAX) were considered as maximal values. Moreover, absolute and percentage oxygen consumption at VT1 and VT2 (V'O_2_ VT1, %V'O_2_ VT1, V'O_2_ VT2, %V'O_2_ VT2), absolute and percentage heart rate (HR VT1, %HR VT1, HR VT2, %HR VT2), and blood lactate concentration (Lactate VT1, Lactate VT2) were examined to compare athletes at the same physiological relative intensities. The time at which the first and second ventilatory thresholds occurred (Time VT1, Time VT2) was also considered. Finally, to compare athletes at the same absolute mechanical intensity, absolute and relative oxygen consumption was measured at 3° and 6° of treadmill incline (V'O_2_ 3°, %V'O_2_ 3°, V'O_2_ 6°, %V'O_2_ 6°), absolute and relative heart rate (HR3°, %HR3°, HR6°, %HR6°), blood lactate (Lactate3°, Lactate6°), and energetic cost (EC3°, EC6°) were examined. Energetic cost was estimated by considering both the aerobic and anaerobic contribution to metabolic power, as described previously (Zoppirolli et al., 2015).

### Statistical Analyses

**Figure 2:**
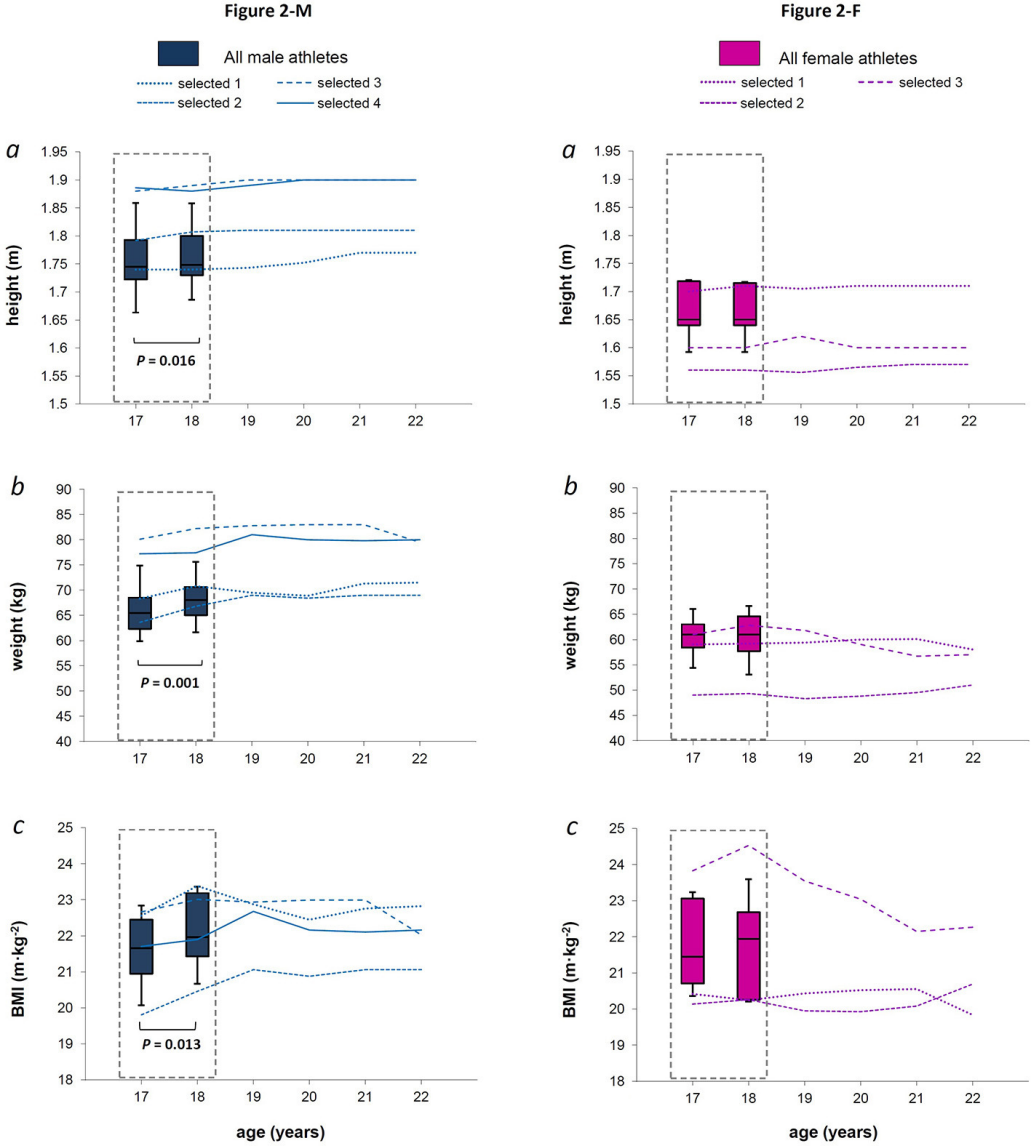
**Figures 2-M and 2-F**. For males and females (Figures 2-M and 2-F, respectively), the box plot represents medians, 9, 25, 75, and 90th percentiles of height, weight, and BMI (panel *a, b*, and *c*, respectively), measured over the late teenage period (data in dotted-lined boxes) in the entire group. The *P*-value refers to the statistical age effect during the late teenage period. In each panel, the line plots represent each selected athlete from 18 to 22 years of age.

**Figure 3:**
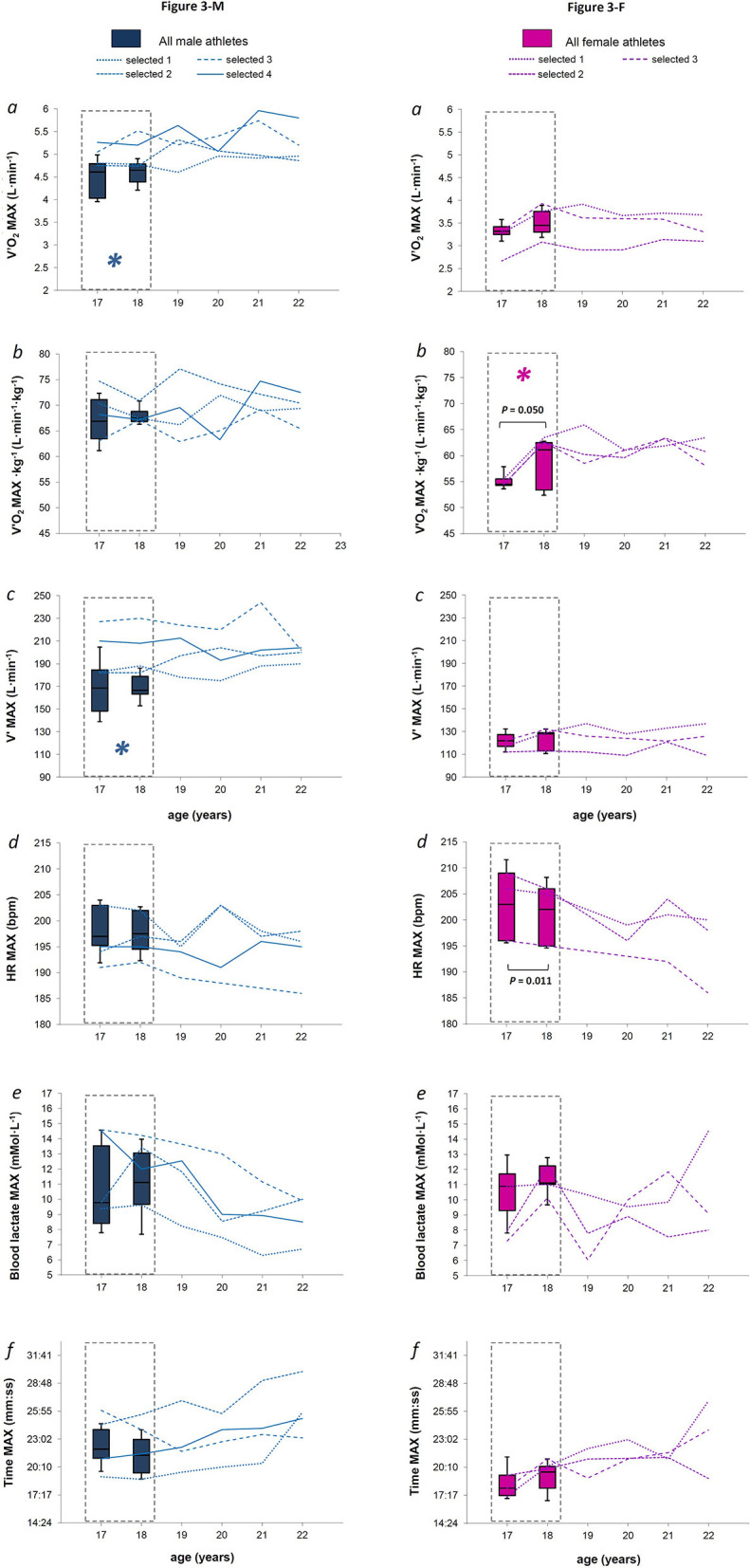
**Figures 3-M and 3-F**. For males and females (Figures 3-M and 3-F, respectively), the box plot represents medians, 9, 25, 75, and 90th percentiles of maximal values of absolute oxygen consumption (panel *a*), mass-scaled oxygen consumption (panel *b*), ventilation volume (panel *c*), absolute heart rate (panel *d*), blood lactate concentration (panel *e*), and test time (panel *f*) measured over the late teenage period (data in dotted-lined boxes) in the entire group. The *P*-value refers to the statistical age effect during the late teenage period. In each panel, the line plots represent each selected athlete from 18 to 22 years of age. *indicates possible parameters early identifying further athletes' selection, when all the criteria presented in the statistical analysis paragraph were satisfied.

**Figure 4:**
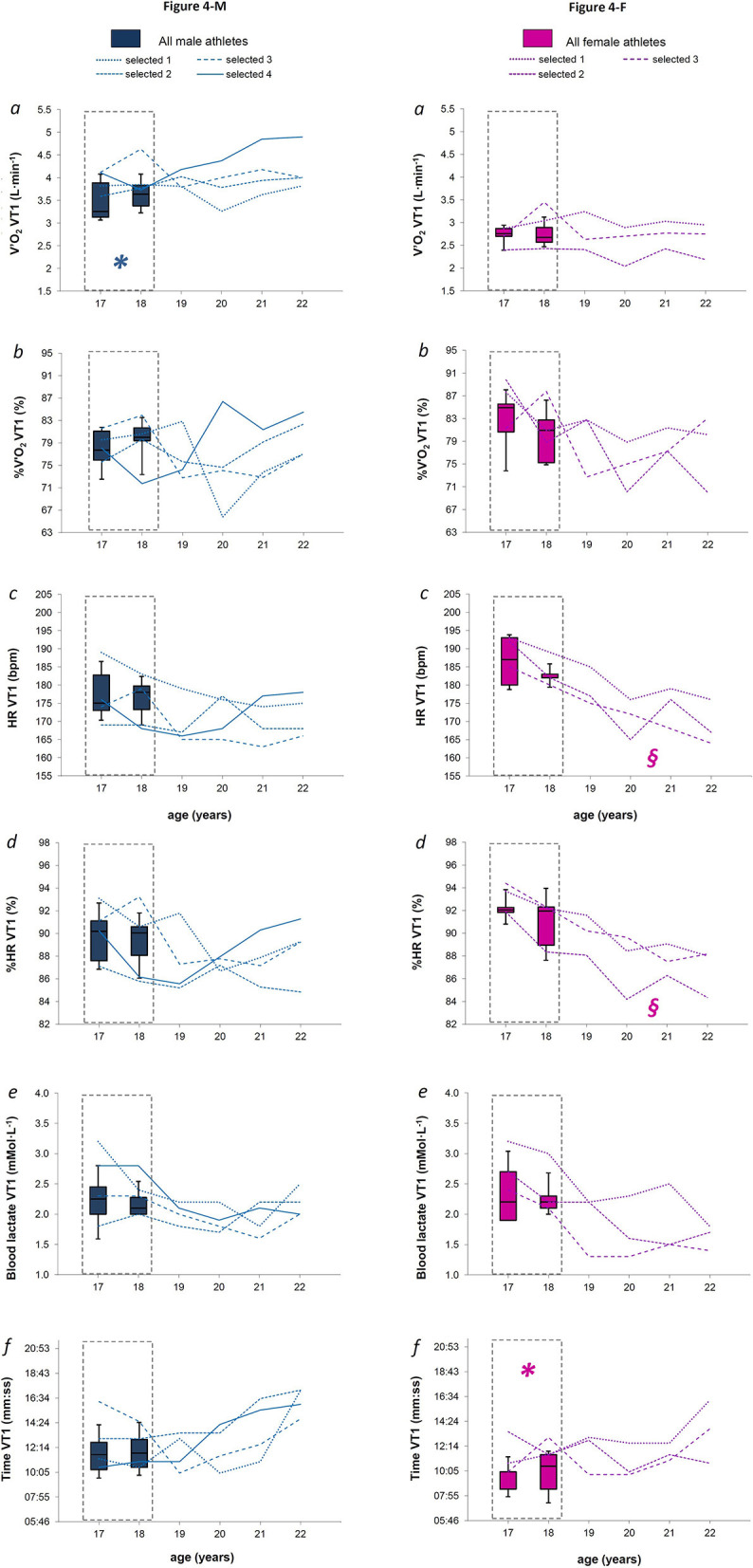
**Figures 4-M and 4-F**. For males and females (Figures 4-M and 4-F, respectively), the box plot represents medians, 9, 25, 75, and 90th percentiles of the values measured at the first ventilator threshold (VT1) for absolute oxygen consumption (panel *a*), relative oxygen consumption (panel *b*), absolute heart rate (panel *c*), relative heart rate (panel *d*), blood lactate concentration (panel *e*), and time of first threshold occurrence (panel *f*) measured over the late teenage period (data in dotted-lined boxes) in the entire group. In each panel, the line plots represent each selected athletes from 18 to 22 years of age. **§** indicates a most likely effect of age after the later teenage period, while * indicates possible parameters early identifying further athletes' selection, when all the criteria presented in the statistical analysis paragraph were satisfied.

**Figure 5:**
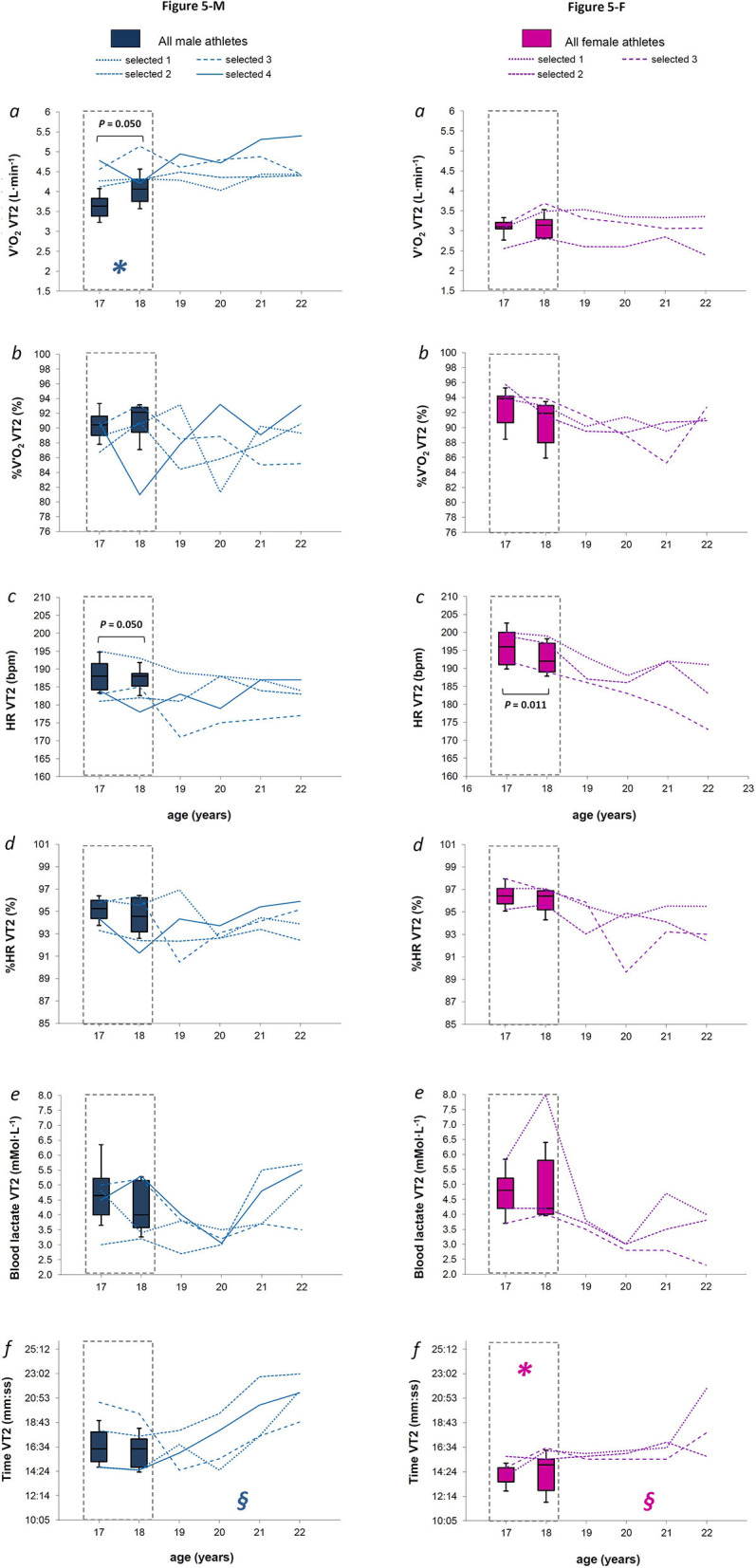
**Figures 5-M and 5-F**. For males and females (Figures 5-M and 5-F, respectively), the box plot represents medians, 9, 25, 75, and 90th percentiles of the values measured at the second ventilator threshold (VT2) for absolute oxygen consumption (panel *a*), relative oxygen consumption (panel *b*), absolute heart rate (panel *c*), relative heart rate (panel *d*), blood lactate concentration (panel *e*), and time of second threshold occurrence (panel *f*) measured over the late teenage period (data in dotted-lined boxes) in the entire group. The *P*-value refers to the statistical age effect during the late teenage period. In each panel, the line plots represent each selected athlete from 18 to 22 years of age. **§** indicates a likely effect of age after the late teenage period, while * indicates possible parameters early identifying further athletes' selection, when all the criteria presented in the statistical analysis paragraph were satisfied.

**Figure 6:**
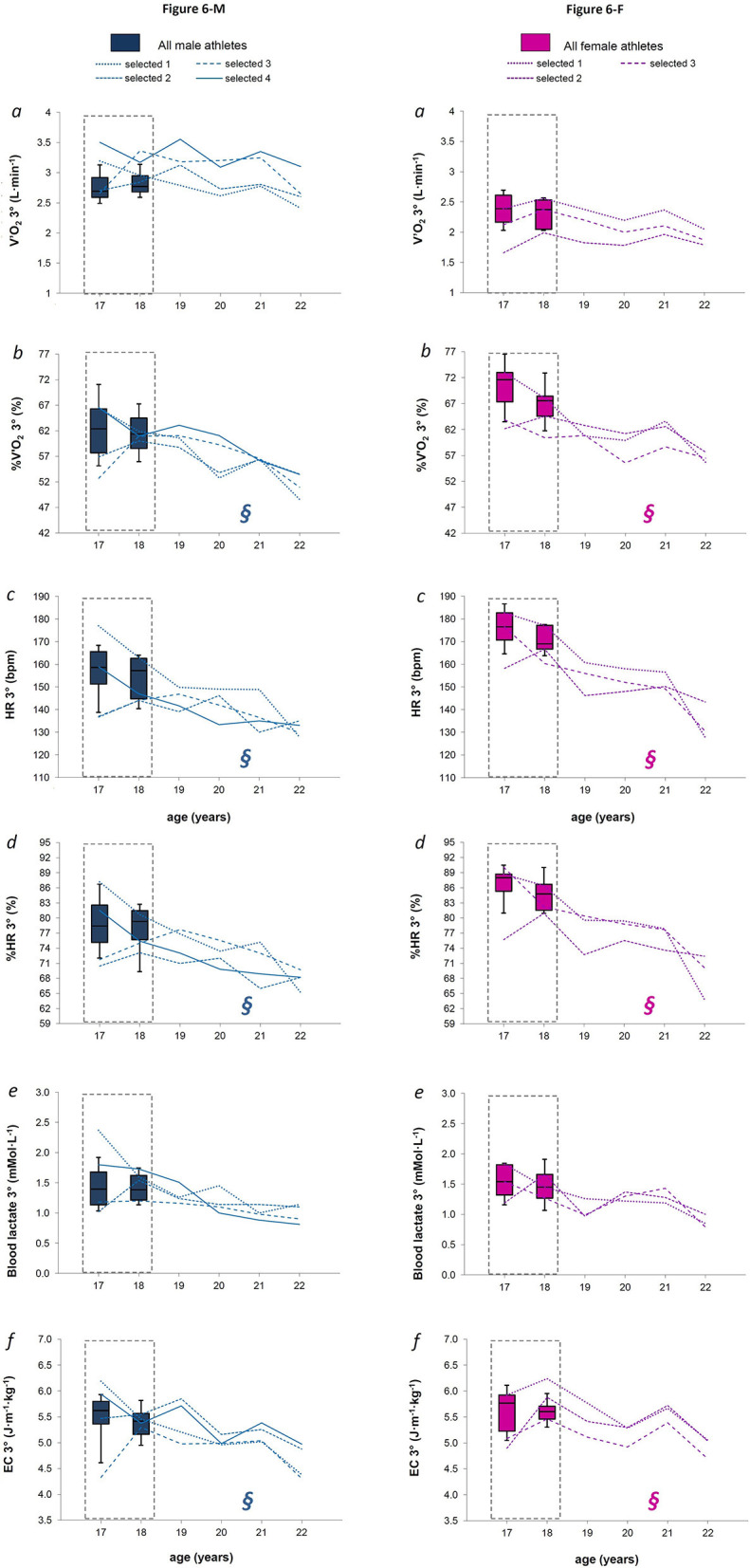
**Figures 6-M and 6-F**. For males and females (Figures 6-M and 6-F, respectively), the box plot represents medians, 9, 25, 75, and 90th percentiles of the values measured at 3° of treadmill incline (3°) for absolute oxygen consumption (panel *a*), relative oxygen consumption (panel *b*), absolute heart rate (panel *c*), relative heart rate (panel *d*), blood lactate concentration (panel *e*), and energetic cost of diagonal skiing (panel *f*) measured over the late teenage period (data in dotted-lined boxes) in the entire group. In each panel, the line plots represent each selected athlete from 18 to 22 years of age. **§** indicates a likely effect of age after the late teenage period.

**Figure 7:**
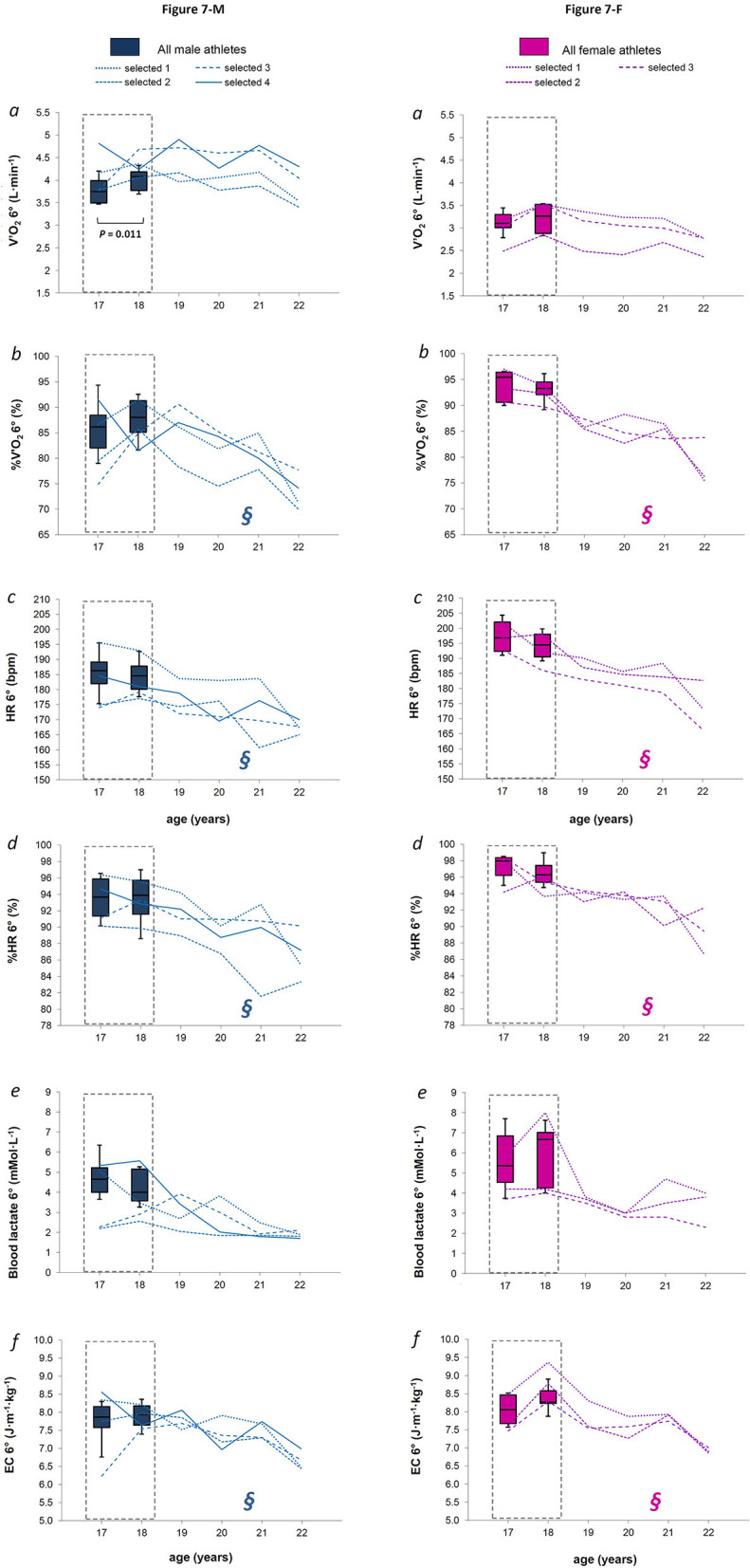
**Figures 7-M and 7-F**. For males and females (Figures 7-M and 7-F, respectively), the box plot represents medians, 9, 25, 75, and 90th percentiles of the values measured at 6° of treadmill incline (6°) for absolute oxygen consumption (panel *a*), relative oxygen consumption (panel *b*), absolute heart rate (panel *c*), relative heart rate (panel *d*), blood lactate concentration (panel *e*), and energetic cost of diagonal skiing (panel *f*) measured over the late teenage period (data in dotted-lined boxes) in the entire group. The *P*-value refers to the statistical age effect during the late teenage period. In each panel, the line plots represent each selected athlete from 18 to 22 years of age. **§** indicates a likely effect of age after the late teenage period.

Due to the low number of subjects and the high number of parameters analyzed, a non-parametric statistical analysis was used. In the text, data are presented as median and 95% confidence intervals (*CI*). In the figures (from Figures 2–**7**), the box plot shows medians of male (-M) and female (-F) groups, with 10, 25, 75, and 90th percentile during the late teenage period. A *Wilcoxon Signed-Rank Test* for paired samples was applied to analyze the effect of age during the late teenage period (between 17 and 18 years old), for both sexes independently. Statistical analysis was carried out using statistical software (SPSS 11.0, SPSS Inc., Chicago, IL, USA) and statistical significance was set at *P* < 0.05.

Moreover, a descriptive analysis was used to evaluate the trend of measured parameters after the late teenage period in M and F selected athletes, independently, as well as to identify possible parameters associated with further selection since the late teenage period in the two sexes. After the late teenage period, a likely increasing or decreasing trend was considered if all the selected athletes showed at least three consecutive increasing or decreasing values from the age of 18 onward, and if all the values measured at the age of 22 were above or below the 75 or 25 percentile, respectively, than the values measured at the age of 18. During the late teenage period, the parameters in the selected athletes that showed values within/above the 75th percentile, or within/below the 25th percentile at both 17 and 18 years of age, were considered as possible characteristics predicting further athlete selection.

## Results

Fourteen M and nine F XC skiers meeting all the inclusion criteria required for the analysis were found. After the late teenage period, four M and three F continued to be evaluated as they were selected for the national team (Figure 1). Figure 2 shows anthropometric characteristics while Figure 3 displays maximal physiological values as well as time to exhaustion data over progressive chronological age, both for males (M) and females (F). Figures 4, 5 show physiological values at VT1 and VT2, respectively, as well as time to reached thresholds in males (M) and females (F). Figures 6, 7 show physiological values at 3° and 6° of treadmill incline, respectively, as well as EC of diagonal skiing at 10 and 9 km·h^−1^ for males (M) and females (F), respectively. In the figures, statistical results for age effect during the late teenage period was indicated.

### Anthropometry and Physiological Values During the Late Teenage Period

Height, weight, and BMI was still raised between 17 and 18 years old in M athletes (*P* = 0.016, = 0.001, and 0.013, respectively), with a median difference of 0.01 m (95% *CI* 0.01), 1.7 kg (*CI* 0.7), and 0.46 kg·m^−2^ (*CI* 0.27), respectively. However, these anthropometric parameters did not change in females [all *P* > 0.05, median difference of 0.00 m (*CI* 0.00), 0.3 kg (*CI* 0.8), and 0.12 kg·m^−2^ (*CI* 0.32), respectively] and did not appear to be linked to further success in both sexes (Figures 2-M and 2-F; panel *a, b*, and *c*, respectively). Concerning maximal values, we did not find any effect of age in the M group (all *P* > 0.05, Figure 3-M). In the F group (Figure 3-F), V'O_2_ MAX·kg^−1^ (Figure 3-F panel *b*) was found to increase with age [*P* = 0.050, median difference of 3.3 mL·min^−1^·kg^−1^ (*CI* 2.8), while HR MAX (Figure 3-F panel *d*) decreased (*P* = 0.011, median difference of −1 bpm (*CI* 1.2)] between 17 and 18 years of age. All selected M athletes showed V'O_2_ MAX (Figure 3-M panel *a*) and V'MAX (Figure 3-M panel *c*) values that were within or above the 75th percentile of the entire male group, at both 17 and 18 years of age, with the selected showing a median value of 4.9 L·min^−1^ (with 0.2 L·min^−1^ *CI*) for V'O_2_ MAX and 198 L·min^−1^ (*CI* 14) for V' MAX, between 17 and 18 years of age. All F selected athletes showed V'O_2_ MAX·kg^−1^ values (Figure 3-F panel *b*) that were within or above the 75th percentile of the entire female group, at both 17 and 18 years of age, with the selected showing a median value of 59.0 mL·min^−1^·kg^−1^ (*CI* 3.6) in that age period.

When considering comparable relative physiological workloads (intensities associated to VT1 and VT2), only V'O_2_ VT2 increased in M athletes between 17 and 18 years of age [Figure 5-M panel *a, P* = 0.050, median difference of 0.12 L·min^−1^ (*CI* 0.21)], while HR VT2 decreased for both sexes [Figures 5-M and 5-F panel *c, P* = 0.050 and *P* = 0.011 for M and F, respectively, with a median difference of −2 bpm (*CI* 1.4) and −3 bpm (*CI* 2.0)]. All selected M athletes showed V'O_2_ VT1 and V'O_2_ VT2 values (Figures 4-M and 5-M, respectively, panel *a*) within or above the 75th percentile of the entire male group, at both 17 and 18 years of age, with the selected ones showing a median value of 3.8 L·min^−1^ (*CI* 0.2) for V'O_2_ VT1 and 4.3 L·min^−1^ (*CI* 0.2) for V'O_2_ VT2. All selected F athletes reached both VT1 and VT2 later than the entire female group, with values of Time VT1 and Time VT2 that were within or above the 75th percentile at both 17 and 18 years of age (Figures 4-F and 5-F, respectively, panel *f*).

When considering specific absolute mechanical intensities (3° and 6° of treadmill incline), only V'O_2_ 6° tended to increase significantly between 17 and 18 years of age in M skiers [Figure 7-M, panel *a*; *P* = 0.011 with a median difference of 0.26 L·min^−1^ (*CI* 0.18)]. In both sexes, any physiological parameters measured at specific absolute mechanical intensities were likely to discriminate against further talent selection (Figures 7-M and 7-F).

### Anthropometry and Physiology After the Late Teenage Period in Selected Athletes

Anthropometric parameters as well as physiological values measured at maximal stages apparently did not change after the late teenage period in selected athletes of both sexes (Figures 3-M, 3-F, 4-M, and 4-F). HR VT1 and %HR VT1 showed a likely decreasing trend in F selected athletes after the late teenage period (Figure 4-F, panel *c* and *d*, respectively), while Time VT2 showed a most likely increasing trend in both M and F selected athletes after the late teenage period (Figures 5-M and 5-F, respectively, panel *f*). Many meaningful effects of age apparently occurred in selected athletes of both sexes when analyzing the physiological values elicited by specific absolute mechanical intensities after the late teenage period (Figures 6-M, 6-F, 7-M, and 7-F). A most likely decreasing trend was observed for EC 3° and EC 6° in both sexes (Figures 6-M, 6-F, 7-M, and 7-F, panel *f)*. In both sexes, a likely decreasing trend in %V'O_2_, HR, and % HR at both 3 and 6° of treadmill incline (Figures 6-M, 6-F, 7-M, and 7-F, panel *b, c*, and *d*, respectively) also occurred.

## Discussion

Our investigation aimed to longitudinally analyze anthropometric and physiological characteristics in high-level junior XC skiers during the late teenage period, and in selected XC skiers after the late teenage period. Moreover, we aimed to observe “*a posteriori”* which characteristics during the late teenage period could predict further talent selection. The main findings were that anthropometric characteristics were still raised between 17 and 18 years in males, while absolute and relative physiological characteristics at maximal stages, did not change significantly. Absolute V'O_2_ at VT2 and moderate sub-maximal intensity increased significantly, while HR at VT2 decreased. In females between 17 and 18 years of age, anthropometric characteristics did not change, while some physiological values at maximal and sub-maximal stages developed significantly (i.e., V'O_2_ MAX ·kg^−1^ increased while HR MAX and HR VT2 decreased). Since the late teenage period, the selected males showed high values of absolute (i.e., L·min^−1^) oxygen consumption and ventilation volumes at maximal stages, as well as high values of absolute oxygen consumption at VTs intensities with respect to the entire group. Since the late teenage period, the selected females showed high values of mass-scaled (i.e., mL·min^−1^·kg^−1^) oxygen consumption as well as a later occurrence of VTs with respect to the entire group. Finally, in selected athletes of both sexes a significant decrease in the energetic cost of skiing was observed after the late teenage period at sub-maximal intensities, together with significant reductions in oxygen consumption and heart rate values.

Even though we did not monitor athletes' training habits or race results, we could compare some physiological values measured in male and female athletes at the age of 18 years old in the present investigation with previously published data about Swedish elite XC skiers of the same age (Larsson et al., 2002). Considering maximal physiological values, we found that the male athletes here reached average absolute V'O_2_ MAX values of 5.0 ± 0.4 L·min^−1^ (see supplementary material) compared to 5.2 ± 0.6 L·min^−1^ found in the Swedish junior elite, and mass-scaled V'O_2_ MAX values of 68.5 ± 2.1 (see supplementary material) compared to 71.1 ± 7.4 mL·min^−1^·kg^−1^. The female counterparts showed absolute V'O_2_ MAX values of 3.6 ± 0.4 L·min^−1^ (see supplementary material) compared to 3.8 ± 0.2 L·min^−1^ of the Swedish junior elite, and mass-scaled V'O_2_ MAX values of 57.3 ± 5.4 (see supplementary material) compared to 59.8 ± 3.2 mL·min^−1^·kg^−1^. These data attest that athletes of both sexes identified by the regional teams to compete in the young national XC skiing circuit, and here examined, had on average a high physiological performance capacity, relative to the specific age considered.

### Longitudinal Analysis During and After the Late Teenage Period

#### Anthropometric Parameters

Male skiers showed a significant increase in height, weight, and BMI values between 17 and 18 years [median difference of 0.01 m (*CI* 0.01), 1.7 kg (*CI* 0.7), and 0.46 kg·m^−2^ (*CI* 0.27), respectively], reaching median anthropometric values of 1.74 m (0.04 m *CI*), 68.0 kg (3.2 kg CI,) and 22 kg·m^−2^ (0.36 kg·m^−2^ *CI*) for height, weight, and BMI, respectively (Figure 2-M) at 18 years of age. This increasing trend is in line with the still ongoing maturation process in males at that age (Cacciari et al., 2006), confirmed to be valid not only for the general population but also for elite endurance athletes (Steiner et al., 2019). On the other hand, female athletes in our study demonstrated an already completed growth process at 17 years of age, attesting their median anthropometric values at 1.65 m (0.03 m *CI*), 61.0 kg (3.8 kg *CI*), and 21.7 kg·m^−2^ (0.9 kg·m^−2^ *CI*), for height, weight, and BMI, respectively (Figure 2-F). It is well-known that physical maturation process in female adolescents ends earlier than in their male counterparts (Cacciari et al., 2006), due to the anticipated peak growth velocity and development process. Also in males, anthropometric parameters showed a stabilization after the late teenage period, accordingly to general population normative data (Cacciari et al., 2006).

#### Physiological Parameters at Maximal Exercise Intensity

Absolute and mass-scaled V'O_2_ MAX were constant between 17 and 18 years in male skiers (Figure 3-M, panel *a*). On the other hand, mass-scaled V'O_2_ MAX increased significantly in females in that period (median difference of 6.6 mL·min^−1^·kg^−1^) (Figure 3-F, panel *b*). Even though a significant increase in V'O_2_peak or V'O_2_ MAX was observed in both untrained subjects or endurance athletes during pre-puberty and adolescence (Paterson et al., 1987; Naughton et al., 2000; Bjerring et al., 2019; Steiner et al., 2019), it was shown that the rate of V'O_2_ MAX is highest around the age of the peak height velocity (12 and 14 years old for females and males, respectively), thus constantly decreasing over years, with V'O_2_ MAX values approaching a plateau around the end of the teenage period in both sexes (Geithner et al., 2004). This was also confirmed for adolescent male XC skiers (Rusko, 1987, 1992). On the other hand, the significant increase in mass-scaled V'O_2_ MAX here observed in female athletes during the late teenage period is sustained by two previous studies indicating a substantial increase in sport-specific V'O_2_peak as a consequence of various training interventions between 16 and 19 years old (Skattebo et al., 2016; Carlsson et al., 2017). Altogether, these data suggest greater adaptations for maximal physiological values in female than in male XC skiers during the late teenage period, presumably due to a different training level at their starting point.

When analyzing the data of the athletes selected by the national team, we found non-appreciable changes in neither absolute nor relative V'O_2_ MAX after the late teenage period, in both sexes (Figures 3-M and 3-F, panel *a* and *b*). Even though residual possibilities for a further increase of V'O_2_ MAX after 20 years of age have been previously reported for international-level XC skiers (Rusko, 1987, 1992), the low number of selected athletes in our study, together with the type of testing protocol proposed to the athletes (it may not be optimal to measure maximal physiological values because of increments in slope only Midgley et al., 2008) could partially explain the lack of further V'O_2_ MAX improvement after the late teenage period in the selected athletes. However, elite adult male XC skiers also reported insignificant changes in V'O_2_peak over the preparation and competition phases of one XC skiing season (Losnegard et al., 2013).

We did not find any significant effect of age on maximal blood lactate concentration, maximal ventilation volumes, and time to exhaustion across and after the late teenage period in both sexes (Figures 3-M and 3-F, panel e, c and f respectively). It was previously reported that maximal ventilation increases significantly across age in endurance athletes aged between 10 and 15, as an effect of physical growth (Mercier et al., 1991). As our subjects were near the end of their maturation process, it is reasonable that they had already reached a stable trend for maximal ventilation during the late teenage period.

#### Physiological Parameters at Sub-Maximal Exercise Intensities

When considering sub-maximal intensities, we found that absolute oxygen consumption at VT2 and moderate sub-maximal exercise intensity increased during the late teenage period in male athletes, in relation to the progressive increase of anthropometric values, while it remained stable in females, as did the anthropometric parameters. Interestingly, a significant decrease of absolute heart rate values was found at the VT2 intensity, maybe as a consequence of endurance training. However, neither blood lactate nor exercise economy changed across the late teenage period in both sexes. Sparse literature describes longitudinal development of physiological parameters related to exercise performance during adolescence (Bragada et al., 2010). When considering previous training studies on junior XC skiers, some investigations showed no changes in skiing economy as a consequence of different training regimes in athletes of this age (Skattebo et al., 2016; Carlsson et al., 2017), while other did (Hoff et al., 1999, 2002).

When analyzing the data of the selected athletes after the late teenage period, it was noted that some parameters changed significantly in both sexes over the years (Figures 4-M, 4-F, 5-M, 5-F, 6-M, 6-F, 7-M, and 7-F). A likely decrease in absolute and relative heart rates was found in male and female athletes at both absolute sub-maximal exercise intensities, and at the VT1 intensity in females, suggesting an increased efficiency of cardiovascular functions after the late teenage period in high-level XC skiers. It was previously reported that endurance training positively influences the growing of both right (D'Ascenzi et al., 2017) and left (Bjerring et al., 2019) heart sides from an early age (10–15 years old) without influencing systolic and diastolic functions at those ages, with respect to sedentary young people (D'Ascenzi et al., 2017; Bjerring et al., 2019). In adolescents (13–19 years old), long-term endurance training (30 min, at least five times a week for at least 2 years) induces both heart structural changes and few functional changes at rest (Rundqvist et al., 2017), revealing that physiological cardiac remodeling occurs due to long-term endurance exercise from early adolescence (Csecs et al., 2020), taking also functional improvements thereafter (Poh et al., 2008).

Also the energetic cost of skiing decreased unequivocally after the late teenage period in selected athletes of both sexes, at both low and moderate exercise intensities. This, together with a decreasing trend for oxygen consumption, indicated a more efficient aerobic energy production at low but also at moderate absolute mechanical intensities. This was in line with a previous study showing how skiing economy improves during the course of preparation and competition phases of a XC skiing season in elite adult athletes (Losnegard et al., 2016), as consequence of physiological and biomechanical improvements due to the great amount of general and specific training. As expected, the time of VT2 occurrence showed a likely increasing trend after the late teenage period in selected athletes, while time for VT1 occurrence did not show an appreciable trend. However, only heart rate at VT1 in females seemed to decrease after the late teenage period, while the rest of the physiological parameters measured at VT1 (oxygen consumption and blood lactate) and VT2 (oxygen consumption, heart rate, and blood lactate) did not show an evident development across years. An early report showed no differences over adolescence for the percentage of V'O_2_ MAX measured at anaerobic threshold in well-trained adolescent XC skiers (Rusko, 1987). Some recent investigations confirmed stable physiological values related to VTs in high-level endurance athletes throughout a training season (Lucia et al., 2000; Polat et al., 2018), as already seen for maximal values. Complete physical maturation together with the high level of performance of our athletes since the late teenage period justifies our findings.

### Parameters That Characterize Successful Athletes at an Early Stage

Some useful information can be drawn by our data, even though all the stages of talent recognition and nurture are very challenging due to the complex systems surrounding the athletes. Genetics, motivation and attitude to exercise, sport environment, family support, stress resilience, residual trainability, and recovery from injuries all contribute to determine all the phases of this process, from talent detection to talent development (Vaeyens et al., 2009; Issurin, 2017). Sometimes an analysis based on anthropometric and physiological parameters might not be adequate in discriminating during adolescence, athletes that performed successfully at an elite level after the late teenage period, found a predictive prevalence of psychological attributes (Issurin, 2017).

As demonstrated in the previous sections, the athletes monitored here were, on average, of a high level during their late teenage period from a physiological perspective, when compared to the data available in the literature (Larsson et al., 2002). Thus, we have analyzed talents already identified in early stages of their sport career, and developed under the guidance of the regional teams. Unfortunately, to our knowledge no literature is available about talent identification or selection in young XC skiers, and finding an interpretation supporting our data is challenging. However, we can compare “*a posteriori*” the characteristics of selected athletes with the data of the entire group during the late teenage period, trying to identify possible anthropometric or physiological parameters that characterize elite XC skiers at an early stage.

In both sexes, anthropometric parameters seemed to fail in discriminating promising athletes during the late teenage period (Figures 2-M and 2-F). These results are in line with the study of Larsson et al. (2002) that found no significant relationships between anthropometry and performance level in elite junior XC skiers of 18 years old (Larsson et al., 2002). Our results are also partly in line with a longitudinal analysis of national-level junior speed-skaters monitored between 17 and 21 years of age (de Koning et al., 1994), where no differences in anthropometric and physiological variables measured during the late teenage period were found between athletes who were successful or unsuccessful after the late teenage period, making it difficult to distinguish further talent after the late teenage period.

Absolute and mass-scaled V'O_2_ MAX, for males and females, respectively, and maximal ventilation volumes for males were found to be possible parameters during the late teenage period for the discrimination of those who were subsequently selected by the national team from those who were not (Figures 3-M and 3-F) since all the selected athletes showed values within or above the 75th percentile of the entire group at both 17 and 18 years of age. Mass-scaled V'O_2_ MAX failed in discriminating the two groups during the late teenage period, even though the body mass of selected athletes did not apparently differ from the rest of the group. Probably, the increasing trend found in anthropometric parameters of males during the late teenage period still acted as a confounding factor, indicating absolute V'O_2_ MAX as a more valid indicator of performance in males. However, it was previously shown that absolute V'O_2_ MAX values for males (Larsson et al., 2002; Sandbakk et al., 2010b, 2011) and mass-scaled V'O_2_ MAX values for females (Larsson et al., 2002; Sandbakk et al., 2010b, 2011) are better related to modern XC skiing performance. When considering previous literature concerning national to world-class level XC skiers, it was shown that absolute V'O_2_ MAX while roller-skiing to exhaustion ranged from 4.7 ± 0.7 to 5.2 ± 0.5 L·min^−1^ in male juniors of 18 years old, depending on the technique used (Larsson et al., 2002; Sandbakk et al., 2011). Further in an athlete's career (between 23 and 25 years), V'O_2_ MAX values of 5.5 ± 0.3 to 5.9 ± 0.5 L·min^−1^, and maximal ventilation values from 192± 20 to 204 ± 14 L·min^−1^ were found in male elite XC skiers, depending on the technique and protocol used (Sandbakk et al., 2010b; Losnegard et al., 2013; Andersson et al., 2014). These data confirmed that a combination of high values of absolute oxygen V'O_2_ MAX together with high maximal ventilation volumes are features that describe high-level male XC skiers.

Few of the physiological parameters measured at the VTs intensities or at sub-maximal mechanical intensities (3 and 6°, respectively) were found to possibly discriminate further talent from an early age (Figures 4-M, 4-F, 5-M, and 5-F). Specifically, all the selected males showed higher absolute oxygen consumptions at VTs, while all selected females showed a later occurrence of VTs than the entire correspondent group. These findings underlined the importance of aerobic capacities in predicting further performance in XC skiers of both sexes and are supported by previous data about elite junior XC skiers. Indeed, significant correlations were found between absolute oxygen consumption at OBLA or second ventilatory threshold and XC skiing performance in male athletes (Larsson et al., 2002), or when analyzing the two sexes together (Sandbakk et al., 2011). Also at these intensities, absolute rather than mass-scaled oxygen consumption better correlated with XC skiing performance (Larsson et al., 2002; Sandbakk et al., 2011). All these findings indicate an advantage for heavier XC skiers in modern XC skiing performance, due to the elevated requirements of speed and power outputs.

Contrary to what was hypothesized, skiing economy did not result in a discriminating factor between groups (Figures 2 *e,f*, 3 *e,f*), in both sexes. The available literature indicated that skiing economy was one of the most important determinants of XC skiing performance, being able to distinguish high-level from recreational XC skiers (Ainegren et al., 2013; Zoppirolli et al., 2015) and world-class from national-class athletes (Sandbakk et al., 2010a,b), but also being related to differences in performance capacity within homogeneous groups (Millet et al., 2002; Sandbakk et al., 2013). However, the available data are about XC skiers older than 23 years, while no studies analyzed the longitudinal development in skiing economy in younger XC skiers. From our results, skiing economy is most likely not indicative of further XC skiing performance in high-level adolescent athletes, but it decreases significantly after the late teenage period in selected athletes. This result is in line with a longitudinal analysis on junior speed-skaters (de Koning et al., 1994) which found no difference in mechanical efficiency during the late teenage period between athletes that were or not successful after the late teenage period.

The present study reported a first attempt to evaluate which anthropometric and physiological parameters measured during the late teenage period could help discriminate further talent, in junior XC skiers. Here we analyzed athletes' physiological characteristics during an incremental test to exhaustion performed with the diagonal-stride technique (both upper and lower body involved in the propulsive actions Andersson et al., 2014). However, other types of testing protocols might improve the process of talent selection in junior XC skiers, since strength, power, and technical capacity are strictly linked to modern XC skiing performance, in addition to endurance capacity (Alsobrook and Heil, 2009) in junior XC skiers (Carlsson et al., 2017). It is known that the higher the amount of upper-body involvement in different XC skiing sub-techniques (diagonal-stride vs. double-poling sub-techniques, where the upper body is primarily involved in the propulsion Holmberg et al., 2005), the higher the difference in endurance performance between sexes (Sandbakk et al., 2012a,b), with females underperforming because of their lower upper body muscle mass, strength, and power with respect to male athletes. Thus, the inclusion of standardized upper body strength and power tests, the evaluation of incremental test to exhaustion performed with the double-poling technique, together with the assessment of psychological attitudes might reveal additional useful information for the talent selection process of XC skiers in the future.

## Limitations

This data analysis represents one of the first attempts to analyze talent development and selection in young high-level cross-country skiers. A high number of inclusion criteria were necessary to perform this peculiar observational study, thus reducing the number of athletes included in the data analysis. Even though our findings fit well with some concerns provided by previous literature, these outcomes are difficult to generalize and further research is needed to deepen these aspects about performance on young athletes.

## Conclusions

For the first time, this study presented a longitudinal analysis of anthropometric and physiological characteristics from the junior to senior level (6 consecutive competitive years) in XC skiers. We found that anthropometric parameters were still raised between 17- and 18-year-old males, while females had already ended their growth process before that age. Most of the absolute and relative physiological parameters at maximal stages did not change significantly during the late teenage period in both sexes, except for V'O_2_ MAX and maximal heart rate which increased and decreased, respectively, in females. At sub-maximal exercise intensity, we found that V'O_2_ at VT2 or moderate exercise intensity increased during the late teenage period in males, while HR at VT2 decreased in both sexes. A progressive decrease in the energetic cost of skiing was found after the late teenage period in selected athletes of both sexes, in relation to improvements of the aerobic pathway. Even though genetic, environmental, and psychological factors were demonstrated to be of particular importance for exceptional athletic performances, we found that some physiological parameters measured during the late teenage period (thus well over the occurrence of peak maturation velocity) could help in discriminating further performance capacity in high-level XC skiers. Since the late teenage period, high V'O_2_ at maximal stages as well as at the ventilator thresholds' intensity, together with elevated maximal ventilation volumes characterized the male athletes that were further selected to compete internationally. Finally, mass-scaled V'O_2_ at maximal stages together with postponed ventilatory thresholds' occurrence appeared to be good indicators of further talent in females.

The present study can be considered as a pioneering analysis of talent development and selection in XC skiing. More research should be considered over the next few years and an international board should be created to deepen this matter. A multilateral approach should be considered in the future, both from a performance and a psychological perspective. Strength and power tests for upper body and/or maximal tests using the double-poling technique (relying more on upper body work) could reveal other or better predictors, especially in female athletes. Moreover, evaluations of exercise motivation, stress resilience, and residual trainability should be considered in the future when analyzing talent development and selection in young XC skiers, because of the importance of psychological attributes for exceptional sport performances.

## Data Availability Statement

All datasets generated for this study are included in the article/supplementary material.

## Ethics Statement

The studies involving human participants were reviewed and approved by CARU, University of Verona. Written informed consent to participate in this study was provided by the participants' legal guardian/next of kin.

## Author Contributions

CZ was the head of the research and draft the manuscript. All the authors were involved in the data acquisition, processing, analysis, and collaborated in preparing the text of the manuscript.

## Conflict of Interest

The authors declare that the research was conducted in the absence of any commercial or financial relationships that could be construed as a potential conflict of interest.
